# Electrochemistry/Photoelectrochemistry-Based Immunosensing and Aptasensing of Carcinoembryonic Antigen

**DOI:** 10.3390/s21227742

**Published:** 2021-11-21

**Authors:** Jingjing Jiang, Jili Xia, Yang Zang, Guowang Diao

**Affiliations:** School of Chemistry and Chemical Engineering, Yangzhou University, Yangzhou 225002, China; jjj@yzu.edu.cn (J.J.); jlxia2@163.com (J.X.); gwdiao@yzu.edu.cn (G.D.)

**Keywords:** electrochemistry, photoelectrochemistry, immunosensor, aptasensor, CEA

## Abstract

Recently, electrochemistry- and photoelectrochemistry-based biosensors have been regarded as powerful tools for trace monitoring of carcinoembryonic antigen (CEA) due to the fact of their intrinsic advantages (e.g., high sensitivity, excellent selectivity, small background, and low cost), which play an important role in early cancer screening and diagnosis and benefit people’s increasing demands for medical and health services. Thus, this mini-review will introduce the current trends in electrochemical and photoelectrochemical biosensors for CEA assay and classify them into two main categories according to the interactions between target and biorecognition elements: immunosensors and aptasensors. Some recent illustrative examples are summarized for interested readers, accompanied by simple descriptions of the related signaling strategies, advanced materials, and detection modes. Finally, the development prospects and challenges of future electrochemical and photoelectrochemical biosensors are considered.

## 1. Introduction

As a malignant tumor originating in epithelial tissue, cancer has become a serious global disease that severely threatens human health and lives [1]. Tumor biomarkers are related to the occurrence and development of some cancers and can be applied to the early screening, diagnosis, and prognosis of cancer [2,3]. Among them, carcinoembryonic antigen (CEA), a set of glycoproteins highly relevant to cell adhesion, has been considered as a commonly used cancer biomarker in clinical diagnosis, because its overexpression in human blood serum often means the presence or progression of various diseases such as colorectal cancer, pancreatic cancer, and lung carcinoma [4,5,6,7]. Thus, designing facile and accurate methods for ultrasensitive monitoring of CEA is of crucial importance for saving patient lives and helps to stimulate people’s demands for medical and health services.

To date, several detection methods, including electrochemistry (EC)-, photoelectrochemistry (PEC)-, electrochemiluminescence-, surface-enhanced Raman scattering-, fluorescence-, and chemiluminescence-based immunoassays have been utilized to trace CEA detection in the early diagnosis of various cancers [8,9,10,11,12,13]. Among them, electrochemical and photoelectrochemical biosensors, practical and potential analytical techniques, can convert specific analyte information into readable electrical signals, which achieve high sensitivity, low cost, and a small background in comparison with common optical methods [14,15,16,17]. In particular, the proper bio-chemical probes (e.g., antibodies and aptamers) are composed of powerful affinity interactions and satisfactory binding sites and have been regard as indispensable recognition elements in sensing devices [18,19]. The utilization of antibodies or nucleic acid aptamers not only benefit improved sensitivity and selectivity of CEA biosensors due to the fact of their specific binding sites and desirable designability but also present increased stability and repeatability due to the fact of their easy assembly and stable bioactivity. With the exploitation of new materials and ingenious signaling strategies, the past decade has witnessed the rapid evolution of electrochemical and photoelectrochemical CEA sensing devices, accompanied by the introduction of versatile immuno- and aptamer-based recognition probes. Thus, according to the difference in target-dependent biorecognition events, the developed CEA biosensors can mainly be divided into two categories: immunosensors and aptasensors, although other recognition elements, such as molecularly imprinted polymers, have also been reported [20].

**Figure 1:**
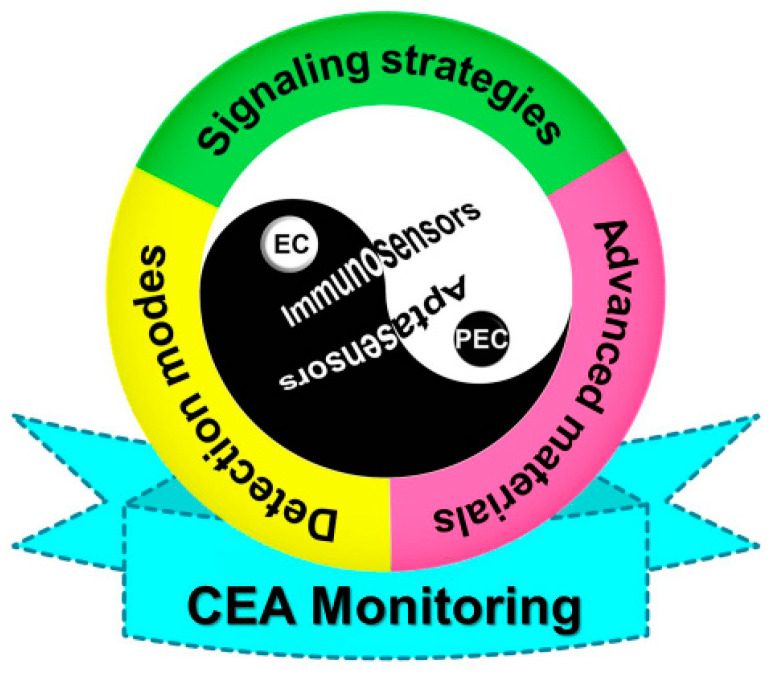
Overview of electrochemistry- and photoelectrochemistry-based immunosensing and aptasensing of CEA.

In this mini-review, we briefly describe the current research status of electrochemical and photoelectrochemical biosensors for CEA assays and focus on recent illustrative immunosensors and aptasensors from the viewpoint of target antibody/aptamer recognition reaction, accompanied by several examples to outline the representative signaling strategies, advanced nanomaterials, and detection modes (Figure 1). Finally, future development trends and challenges are discussed. Moreover, by means of a comprehensive summarization of the newly published achievements in research, we strongly believe that this summarization of EC- and PEC-based CEA aptasensors and immunosensors is of great significance for interested readers and will make a positive contribution to further construction of sophisticated sensing devices due to the increasing enthusiasm of analytical chemists and medical research staff.

## 2. Electrochemistry-Based CEA Detection

### 2.1. Electrochemical Immunosensors

**Figure 2:**
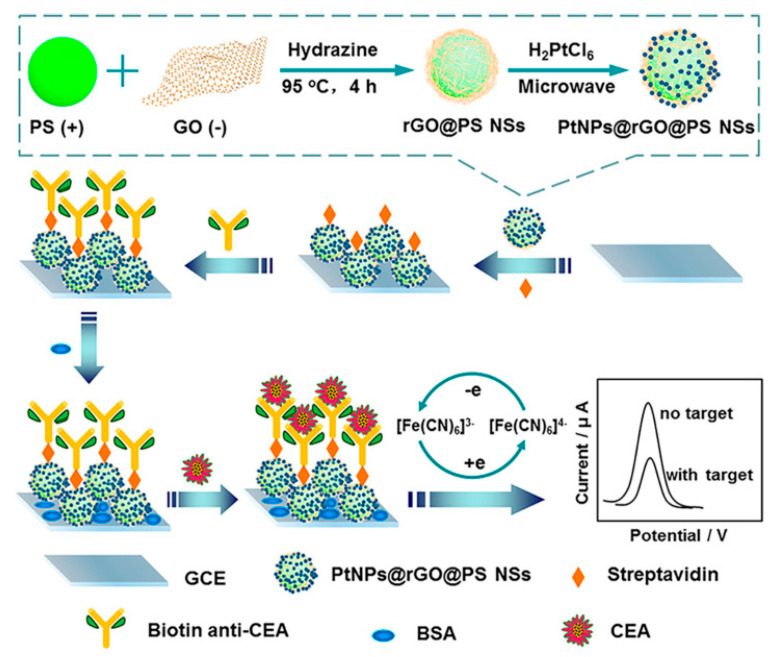
Schematic illustration of the preparation process for PtNPs@rGO@PS NSs and the fabrication of the electrochemical label-free immunosensor. Reprinted with permission from ref. [24]. Copyright 2020 American Chemical Society.

Over the past several decades, immunoassays with specific recognition capability by direct binding of an antigen and its antibody have become a well-established standard detection technique for the quantitative analyses of disease-related biomarkers [21,22,23]. Some recently reported electrochemical immunosensors for CEA determination are listed in Table 1. For example, Yang’s group constructed a novel label-free electrochemical CEA immunosensing platform on the basis of platinum-nanoparticle-decorated reduced-graphene oxide@polystyrene nanospheres (PtNPs@rGO@PS NSs), which were prepared by the hydrazine reduction of graphene oxide (GO) on the surface of PS NSs and the subsequent microwave-induced in situ generation of Pt NPs (Figure 2) [24]. As an ideal electrode matrix, PtNPs@rGO@PS NSs are beneficial for the biofunctionalized modification of streptavidin molecules and the further immobilization of biotinylated CEA antibodies (biotin anti-CEA) due to the fact of their satisfactory biological compatibilities and large specific surface areas. The formed nonconductive immune composites after the addition of target CEA can block electron transfer processes of [Fe(CN)_6_]^3−/4−^ probes and, thus, the obtained current response displays a wide linear negative correlation for CEA (ranging from 0.05 to 70 ng mL^−1^).

**Figure 3:**
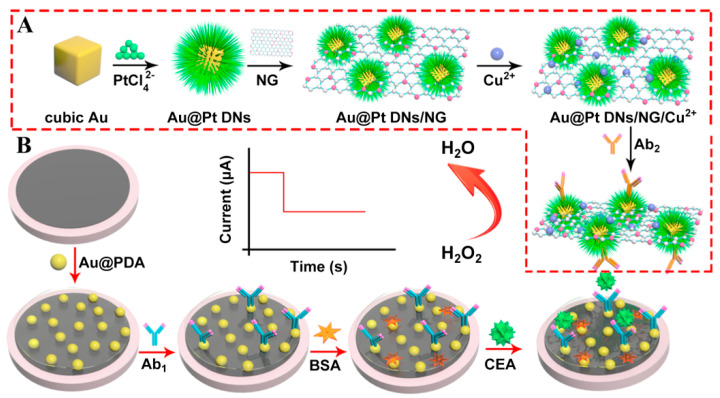
(**A**) The preparation procedures of Au@Pt DNs/NG/Cu^2+^-Ab_2_. (**B**) The fabrication process of the sandwich-type electrochemical immunosensor. Reprinted with permission from ref. [41]. Copyright 2018 Elsevier.

Since the intrinsic features of immune proteins limit their applications in immunoassays as redox partners, the combination of secondary antibodies (Ab_2_) and signal amplification labels has been extensively applied to most immunosensors for the monitoring of current intensities from electroactive probes (e.g., H_2_O_2_ and ferrocene) [37,38]. As a result, it is highly desirable to synthesize effective label materials with excellent catalytic performances in the development of sensitive immunosensing platforms [30,39,40]. Based on this, copper ion-loaded cubic Au@Pt dendritic nanomaterial-functionalized nitrogen-doped graphene (Au@Pt DNs/NG/Cu^2+^) with large surface areas and remarkable adsorption capabilities were first prepared and served as label units to capture numerous Ab_2_ (Figure 3) [41]. Moreover, Au nanoparticle-modified polydopamine (Au@PDA) nanocomposites with superior electronic conductivity as signal transducing units were cast on a glassy carbon electrode (GCE) surface for the immobilization of primary antibodies (Ab_1_). In the presence of target protein, the formation of sandwich-type immune-complex from Ab_1_, CEA, and Ab_2_ can prompt Au@Pt DNs/NG/Cu^2+^ nanocomposites to reach the GCE’s surface and further catalyze the reduction in substrate H_2_O_2_ owing to their peroxidase-like properties. Taking advantage of the synergetic effects of Au@Pt DNs/NG/Cu^2+^ and Au@PDA nanomaterials, a low detection limit (0.167 pg mL^−1^), wide concentration range (from 0.5 pg mL^−1^ to 50 ng mL^−1^), high selectivity, and good practicality were achieved for CEA determination, which may provide a new pathway in clinical analysis and diagnosis.

In recent years, nucleic acid-based immunosensing strategies have been proved as powerful tools for protein identifications via the integration of DNA strands and antibodies to improve analytical performances [42,43]. Among the aforementioned methods, proximity hybridization-assisted amplification generally utilizes a couple of specific antibody-labeled DNA strands (also called proximity probes) to synchronously recognize the target antigen, followed by the hybridization of proximity probes for the stimulation of detection signals [44,45]. According to this consideration, Xiong and coworkers exploited an ultrasensitive electrochemical CEA immunosensor by the intelligent use of proximity hybridization-stimulated, three-layer cascade amplification [46]. Upon addition of the target analyte, the proximity hybridization between two antibody-labeled DNA strands (i.e., Ab-DNA1 and Ab-DNA2) and CEA gave rise to the conformational change of hairpin DNA1 (HP1) and the subsequent degradation process of exonuclease III (Exo III), which resulted in the release of Ab-DNA1:CEA:Ab-DNA2 duplex for Cycle I and the generation of many DNA fragments. The resultant DNA fragments could drive the next catalytic hairpin assembly (CHA, Cycle II) and rolling circle amplification (RCA) processes on the surface of Au electrode through their hybridization reactions towards hairpin DNA2 (HP2). After the RCA process, a large number of guanine-rich long DNA single strands were obtained due to the circular cytosine-rich padlock probes. When the final electrode was immersed in methylene blue (MB) solution, these guanine-rich DNA single strands were successfully linked to numerous MB molecules via the formation of guanine—MB complexes, concomitant with a remarkable peak current enhancement of electroactive MB. A wide linear range over seven orders of magnitude from the direct electrochemical readout indicated that the designed sensing strategy may lay a solid foundation for other biomarker assays.

**Figure 4:**
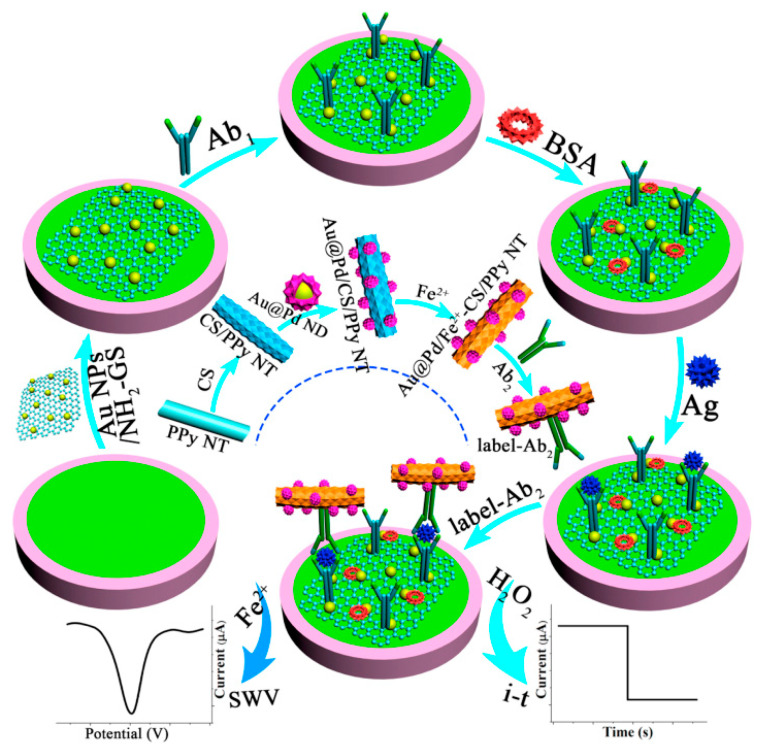
The preparation process of Au@Pd NDs/Fe^2+^-CS/PPy NTs and the schemata of the fabrication process of the working electrode for label-free immunosensors. Reprinted with permission from ref. [47]. Copyright 2018 Elsevier.

Since a large proportion of conventional “off–on” or “on–off” electrochemical biosensors usually only yield one kind of response current, false errors from background noise and environmental influence may lead to unreliable experimental results. A variety of sensing strategies have been established to boost the reliability of analytical data. As shown in Figure 4, a facile sandwich-type immunosensor was developed on account of two electrochemical detection methods [47]. The synthesized amino-functionalized graphene sheet-supported Au nanoparticles (Au NPs/NH_2_-GS) with high electron transfer rate were cast on a GCE surface to constitute the sensing platform. After the target CEA was added, ferrous-chitosan-modified polypyrrole nanotube-supported Au@Pd nanodendrites (Au@Pd NDs/Fe^2+^-CS/PPy NTs) not only acted as efficient electrocatalysts to catalyze the reduction of H_2_O_2_ via the amperometric i–t curve but also served as electroactive probes via the square wave voltammetry (SWV) without additional redox substance. By the comparison of two analytical methods, an overlapped linear concentration range (from 500 fg mL^−1^ to 5.0 ng mL^−1^) was gained, which proved the reliability of this immunosensor and opened an alternative avenue for quantitative monitoring of other tumor markers.

Enlightened by the ratiometric fluorescence and electrochemiluminescence techniques [48,49,50], the construction of dual-potential ratiometric electrochemical biosensors based on the self-calibration of two different current signals is feasible for reducing a number of errors [51,52]. In this communication, using carboxyl-Au nanoparticle-decorated mesoporous CeO_2_ nanoparticles (Au–CeO_2_)-supported toluidine blue (TB) and Au nanoparticle-functionalized Cu_2_S–CuS/graphene (Au–Cu_2_S–CuS/graphene) nanocomposites as signal label and transducing elements, respectively, Wei et al. proposed a novel dual-potential ratiometric electrochemical biosensor to sensitively monitor CEA levels [53]. With the gradual introduction of CEA, the oxidation peak current of TB was enhanced, while the peak current of Cu_2_S–CuS decreased. In addition, the integrated response signal ($\Delta I=\Delta I_{\mathrm{TB}}+\left|\Delta I_{{\mathrm{Cu}}_{2}S-\mathrm{CuS}}\right|$) exhibited a desirable linear positive correlation with a CEA concentration in the range of 0.001–100 ng mL^−1^. Compared with the single signal-based detection mode, the detection limit from ratiometric analysis was much lower than that using $\Delta I_{\mathrm{TB}}$ or $\left|\Delta I_{{\mathrm{Cu}}_{2}S-\mathrm{CuS}}\right|$ as a respective current response. Furthermore, the application of a dual-responsive assay is another strategy to obtain accurate and persuasive detection results by the simultaneous signal outputs from two types of sensing platforms [54,55]. Using electrochemistry-fluorescence dual-responsive methods, the ingenious integration of sensitive electrochemical and stable fluorescent readouts has attracted tremendous interest in recent years. To account for this, a reliable and sensitive electrochemistry-fluorescence dual-responsive immunosensor on the strength of cation exchange reactions was successfully exploited to precisely quantify CEA content [56]. The sandwich-type immune reaction guided the connection of a capture probe (Fe_3_O_4_-Ab_1_), model analyte (CEA), and amplification label (carbon nanotube-poly(amidoamine)-CdSe nanocrystals-Ab_2_, CNT-PAMAM-CdSe NCs-Ab_2_). Upon addition of Ag^+^, thousands of Cd^2+^ can be released through the cation exchange reaction, which achieves the direct detection of Cd^2+^ by electrochemistry and the indirect detection of metal-sensitive dyes (Rhod-5N) with the existence of Cd^2+^ by fluorescence. The combination of CNT–PAMAM materials with large specific surface areas and cation exchange reactions with high exchange efficiencies guarantees a significant improvement in detection sensitivity.

### 2.2. Electrochemical Aptasensors

Aptamers with single-strand DNA or RNA sequences can specifically recognize and bind different kinds of ligand molecules, ranging from small ions to large proteins. With the increasing demand for biological analyses, the appearance of aptamers has shown great potentials in bioassays due to the fact of their obvious superiority in high specificity, simple synthesis, desirable affinity, and long-term storage by comparison with antibodies [57,58,59]. Huang and coworkers employed graphene quantum-dot ionic-liquid nafion (GQDs–IL–NF) nanocomposites and Pb^2+^-assistant cyclic cleavage reaction to construct a novel electrochemical aptasensor for the highly sensitive monitoring of CEA [60]. It is well known that the complicacy of biological matrixes (such as blood serum, human plasma, and urine) can significantly influence the performance of electrochemical CEA biosensors due to the serious nonspecific adsorption [61]. The exploitation of antifouling materials with superior biocompatibility and good chemical durability has been verified as a useful way to overcome this obstacle. For instance, choosing poly(sulfobetaine methacrylate) (PSBMA) and PDA as the antifouling and adhesion substances, respectively, Xu et al. established an electrochemical low-fouling aptasensor in complicated biological matrixes based on the one-step copolymerization of PDA–PSBMA film [62]. During the Michael addition procedure, the CEA aptamer with thiol groups were attached to the surface of PDA–PSBMA/GCE through the covalent binding with PDA. The specific recognition of target CEA and its aptamer could hinder the diffusion of [Fe(CN)_6_]^3−/4−^ towards the electrode surface and cause an obvious suppression of the response current. The low detection limit, down to 3.3 fg mL^−1^, and satisfactory antifouling ability in clinical serum samples confirmed the broad application prospect of antifouling materials.

**Figure 5:**
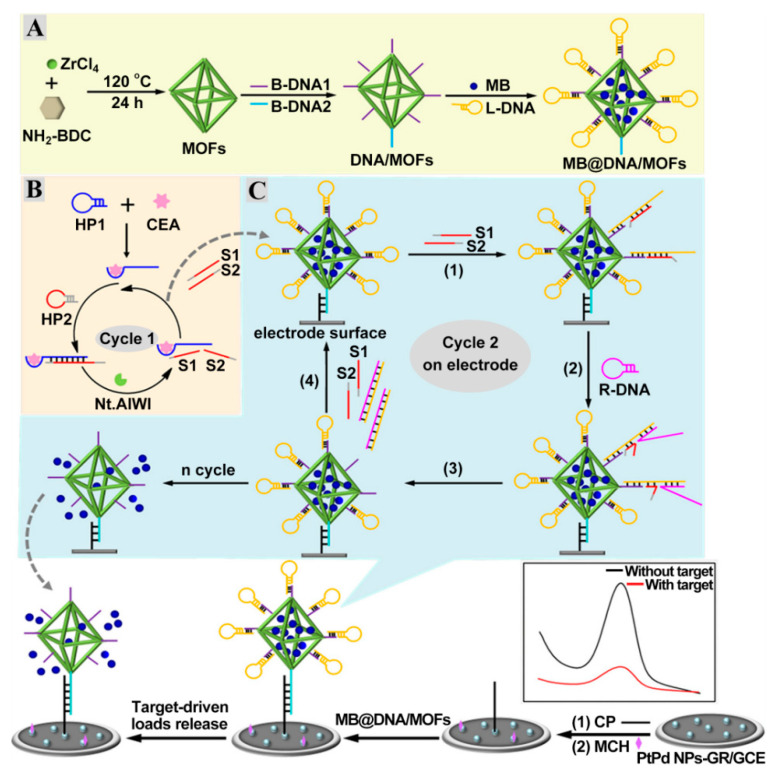
Scheme diagram of the DNA-gated MOF-based electrochemical biosensing platform of CEA. (**A**) Assembly procedure of MB@DNA/MOFs. (**B**) Target-triggered nicking endonuclease cleavage process. (**C**) Signal molecule release from MB@DNA/MOFs on the electrode. Reprinted with permission from ref. [74]. Copyright 2020 American Chemical Society.

Nucleic acid-based amplification technologies, such as strand displacement reaction (SDR) [63,64,65], metal ion-dependent DNAzyme [66,67], hybridization chain reaction (HCR) [68,69,70], and nuclease cycling cleavage [71,72] remarkably enhanced the detection sensitivity. Zhao et al. designed an ultrasensitive impedimetric CEA aptasensor based on the amplification effect of Zn^2+^-dependent DNAzyme-inspired cycling cleavage [73]. The reduction in the substrate DNA’s density on the electrode can effectively circumvent the disadvantages of traditional impedimetric aptasensors (high initial resistance) and, thus, a wide dynamic range from 10 fg mL^−1^ to 10 ng mL^−1^ and an ultra-low detection limit of 7.9 fg mL^−1^ were obtained. However, the involvement of one-dimensional or two-dimensional tracks could restrict their amplification efficiencies. The highly ordered pores from metal–organic frameworks (MOFs) and high structural diversity from DNA self-assembly endow DNA-gated MOFs with the ability of molecular recognition and signal output. As shown in Figure 5A, the surface of MOFs are proactively linked to carboxylated B-DNA1 and B-DNA2 via the amidation interaction and then act as a nanocarrier for the capture of MB and lock DNA (L-DNA) to form three-dimensional tracks (MB@DNA/MOFs) [74]. A hybridization reaction between a capture probe (CP) and B-DNA2 strands triggered the deposition of MB@DNA/MOFs on the electrode, which generated an arresting current signal from the MB. Selecting CEA as a model analyte, the induced nicking endonuclease cycling cleavage by the specific binding of CEA aptamer resulted in the production of numerous S1 and S2 strands (Figure 5B). During the SDR process, the resultant S1 and S2 strands further hybridized with L-DNA on MB@DNA/MOFs to expose toehold segments for the replacement DNA (R-DNA) assembly (Figure 5C). After the liberation of L-DNA strands by the cascade amplification, a large number of MB molecules were released from the pore of MOFs, leading to a reduction in the MB signal. Thus, the sensitive detection of CEA was achieved by the incorporation of target-driven cascade amplification and three-dimensional DNA-gated MOFs.

Owing to the high sequence programmability, abundant DNA nanostructures have been precisely constructed with various sizes and shapes according to Watson–Crick base-pairing rules [75]. A DNA tetrahedron probe, a type of polyhedron, possessed distinct superiorities, including excellent mechanical rigidity and favorable modulating capability, which raised the hybridization efficiencies by the accurate control of probe distances [76]. Herein, Ye’s group put forward a novel double recognition–amplification CEA sensing strategy based on the combination of a DNA tetrahedron and dual-function messenger probes (DMPs) [77]. Using DMP-initiated HCR-induced hemin/G-quadruplex complexes, the quantitative conversion of specific CEA–aptamer recognition events to gaugeable current signals could be realized. The rigid scaffold and ordered orientation of self-assembled DNA tetrahedron probes ensured efficient target accessibility and depressed non-specific adsorption. Under the optimal conditions, this double recognition–amplification aptasensor displayed high sensitivity and selectivity for CEA identification with a wide concentration range from 0.1 pg mL^−1^ to 50 ng mL^−1^ and a low detection limit of 18.2 fg mL^−1^. Analytical performances of other electrochemical CEA aptasensors are summarized in Table 2.

For the past few years, flexible sensors have been emerging as promising candidates to continuously monitor human health circumstances. However, the complicated integration procedures between electrode materials, soft substrates, and current collectors give rise to low stability and weak durability in flexible sensors. To address these problems, the evolution of free-standing electrode-based flexible sensors away from soft substrates and current collectors is urgently needed. As a flexible free-standing electrode, a conducting PPy nanocomposite film with a sandwich structure was prepared by the successive electropolymerization of pentaerythritol ethoxylate-doped PPy (PEE–PPy) and 2-naphthalene sulfonate-doped PPy (2-NS–PPy) [85,86]. After the stepwise modification of Au nanoparticles and CEA aptamers on the thin composite film via electrochemical deposition and Au–S interaction, 6-mercapto-1-hexanol (MCH) was served as a blocking agent to remove the non-specific adsorption. Without the requirement of additional soft substrates and current collectors, this aptamer-functionalized film electrode can be directly applied for the establishment of a flexible free-standing electrochemical CEA aptasensor, which may have promising applications in flexible and wearable electronics.

## 3. Photoelectrochemistry-Based CEA Detection

### 3.1. Photoelectrochemical Immunosensing

**Figure 6:**
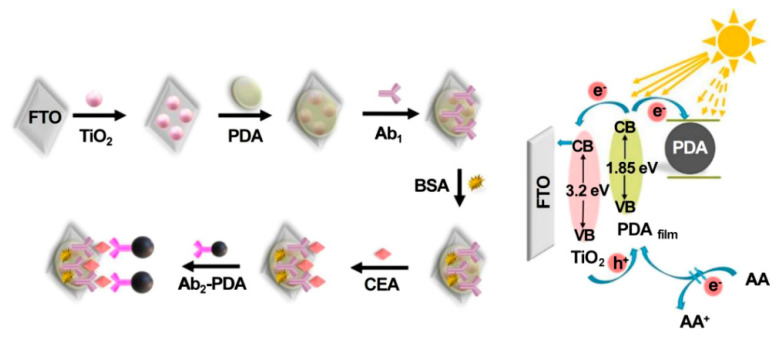
Fabrication and detection mechanism of the PEC immunosensor. Reprinted with permission from ref. [94]. Copyright 2021 American Chemical Society.

Antibodies, commonly produced by several mammals’ immunoreaction against foreign stimuli, have been considered as appealing capture probes or receptors for the detection of various analytes due to the fact of their specific antigen–antibody interaction [87]. With the rapid evolution of sensing strategies and nanomaterials, recent achievements in photoelectrochemical CEA immunosensors brought widespread attention to the early diagnosis of diseases [88,89,90,91]. The example of a label-free photoelectrochemical immunosensing was reported by Wu’s group [92]; in this case, CEA antibody as a typical immunorecognition unit could be covalently linked to the matrix of a CdS nanowire-sensitized WO_3_@BiOI nanocomposite, and the decreased photocurrent was recorded due to the steric insulation from an antibody–CEA immunocomplex. Similarly, depending on the successful preparation of ternary WO_3_/Au/CdS photocatalyst, Zeng et al. developed a sensitive label-free immunosensor for photoelectrochemical CEA assay with a low detection limit of 1 pg mL^−1^ by means of the synergy of the sensitization of CdS and the localized surface plasmon resonance effect of Au NPs [93]. Subsequently, Wu et al. designed a multiple quenching-based immunosensors for CEA detection by using TiO_2_ nanoparticle-sensitized PDA thin film (PDA_film_) as the photoanode and CEA secondary antibody-decorated PDA nanosphere (Ab_2_-PDA) as the signal tag [94]. As shown in Figure 6, after the addition of CEA, Ab_2_-PDA could be linked to the photoanode surface via sandwich immunoreaction. The immobilized PDA nanosphere could not only compete for light absorbance with PDA_film_ and capture the photoelectrons produced from PDA_film_ but also block the access of electron donors to regenerate the corresponding photoactive material due to the formation of steric hindrance, which contributed to the decline of the photocurrent and ensured an excellent sensitivity with a low limit of detection of 40 fg mL^−1^. Moreover, some enzyme-catalyzed signaling strategies were proposed to further improve detection sensitivity [95,96,97]. For instance, Wei et al. developed an ultrasensitive photoelectrochemical immunosensing of CEA based on alkaline phosphatase (ALP)-mediated enzymatic hydrolysis on Cu-doped TiO_2_ composited with carbon nitride [98]. In the presence of the target analyte, the formation of sandwiched immunocomplex enabled ALP–Au-Ab_2_ bioconjugates as enzyme tags to catalyze the generation of ascorbic acid (AA), leading to improvement in the photocurrent response. In another study, Zhang et al. designed an ultrasensitive cathodic immunosensor in which TiO_2_ NP photoanodes enhanced signal output and Ab_1_-decorated Cu_2_O nanowire photocathodes were used for the assembly of Ab_2_-labeled horseradish peroxidase (Ab_2_-HRP) [99]. After the biocatalytic precipitation in the presence of 4-chloro-1-naphtholand and H_2_O_2_, quantitative detection was realized along with the evident photocurrent reduction. This proposed method also indicated that the biorecognition reaction that occurred on the photocathode would present a better anti-interference ability than traditional anodic modification.

**Figure 7:**
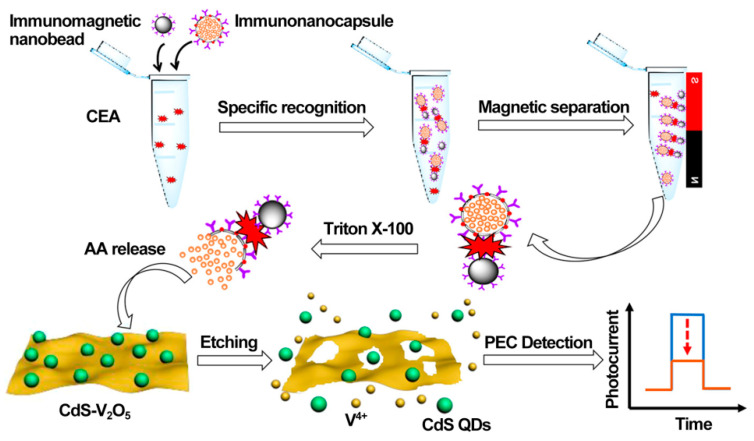
Illustration of the PEC immunoassay for the detection of CEA. Reprinted with permission from ref. [103]. Copyright 2020 American Chemical Society.

**Figure 8:**
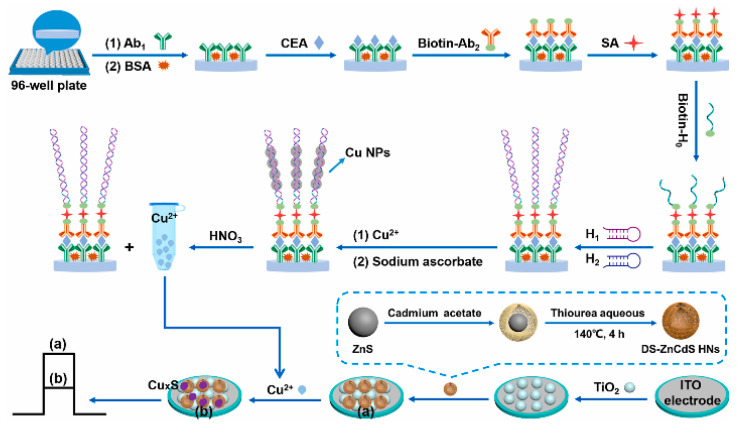
Construction of the designed photoelectrochemical immunosensor for CEA monitoring based on the quenching of HCR-modulated Cu^2+^ sources toward TiO_2_-sensitized DS-ZnCdS HNs. Reprinted with permission from ref. [105]. Copyright 2021 Elsevier.

As a practical design concept, split-type photoelectrochemical sensing strategies have been extensively concerned in the construction of immunosensors because of their inherent advantages such as easy manipulation, simplified electrode modification procedures, and desirable stability [100,101,102]. The split-type sensing platform is composed of two important regions: a signaling transducer region with photoactive materials and a biorecognition region with specific capture probes. Among them, magnetic beads or 96-well plates are generally selected as a matrix for specific biorecognition events, and the photoactive material-modified photoelectrode is chosen as a signal transducer for photocurrent generation, so that the mutual disturbance of both can be eliminated effectively. Based on this, Chen et al. synthesized CdS quantum dot-decorated V_2_O_5_ nanosheets (CdS-V_2_O_5_) and anti-CEA antibody-capped magnetic beads, respectively, and then developed sensitive immunosensing based on AA-dependent acid etching [103]. As displayed in Figure 7, AA-encapsulated liposome immunonanocapsules as the signal tag were bound to the immunomagnetic nanobead surface via CEA-triggered immunorecognition. After magnetic separation, large amounts of AA were released from the captured immunonanocapsule with the aid of Triton X-100, which could effectively etch the V_2_O_5_ nanosheets to V^4+^ via a facile reduction reaction, leading to a significant suppression of the photocurrent response. Following that, Zhu et al. prepared PdPt bimetallic nanozymes-modified CdS nanorods (CdS/PdPt) and constructed a nanozyme-activated split-type photoelectrochemical CEA immunosensor [104]. By using CEA as a model, glucose oxidase (GOx) and Ab_2_-functionalized zeolitic imidazolate framework-8 conjugate was immobilized onto an Ab_1_-decorated 96-well microplate and subsequently catalyzed the oxidation of glucose into H_2_O_2_ as an oxidant. After being mixed with 4-chloro-1-naphthol, the resulting mixture was dropped onto a CdS/PdPt photoanode and catalyzed by PdPt nanozymes to form insoluble precipitates, accompanied by enzymatic bio-etching of CdS nanorods, achieving a synergistically declined output signal with the promotion of detection sensitivity and accuracy. This DNA assembly technique with diverse programmability is a promising candidate for signal amplifications. In this regard, Zang et al. designed a sensitive photoelectrochemical immunosensor by combining HCR-triggered in situ formation of Cu NPs and a Cu^2+^-based quenching reaction [105]. As shown in Figure 8, TiO_2_ and a double-shell ZnCdS hollow nanosphere (TiO_2_/DS-ZnCdS)-modified photoanode was prepared as a signal transducer, and an Ab_1_-functionalized 96-well microplate was fabricated for target capture. In the presence of CEA, biotin-labeled anti-CEA antibodies (biotin-Ab_2_) could be assembled in a 96-well microplate by antigen–antibody interaction and then initiate an HCR reaction to form a long double-stranded DNA (dsDNA) scaffold so that numerous Cu^2+^ ions were adsorbed and, in situ, generated Cu NPs by facile reduction reaction. After acid dissolution, the obtained Cu^2+^ ions could largely suppress the photocurrent due to the formation of Cu_x_S, exhibiting a wide linear range and low detection. These examples suggested that the split-type photoelectrochemical immunosensors could not only dispense with complex electrode modification for improved reproducibility and accuracy but also tend to introduce various signal amplifications for high sensitivity.

**Figure 9:**
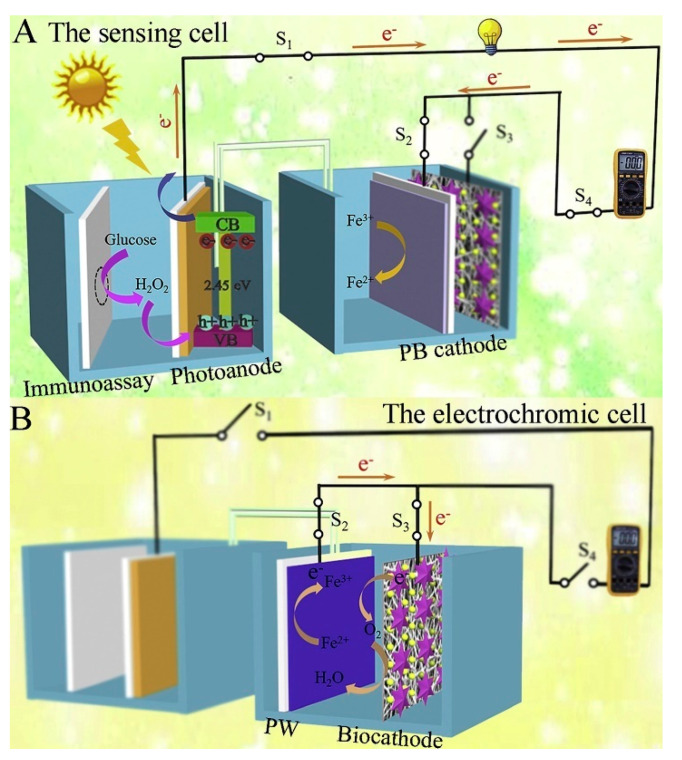
Analytical principle of the salt bridge-connected electrochromic PEC immunosensor with DMM readout: (**A**) The sensing cell; (**B**) The electrochromic cell. Reprinted with permission from ref. [109]. Copyright 2020 Elsevier.

In addition to the above, ongoing efforts have been made to develop other advanced photoelectrochemical immunosensors with the exploitation of new materials and innovative detection modes. Based on this, Wang et al. synthesized an ionic liquid-functionalized metal–organic framework for in situ growth of Au NPs to obtain a Yb–MOF@Au-NP nanocomposite and then constructed a near-infrared light-driven photoelectrochemical CEA immunosensor [106], which benefited from biological detection even in in vivo analysis, because the near-infrared light possessed lower-energy photons and a deeper penetration depth compared to visible light and ultraviolet light. The introduction of Au NPs significantly raised the photocurrent response, four-fold over the pristine Yb-MOF. After the assembly of anti-CEA antibodies, CEA could be linked to the Yb–MOF@Au-NP surface, resulting in a declined signal response. Similarly, Fu et al. synthesized the aggregation of perylene tetraformic acid derivatives (PTCs) as an electron donor–acceptor organic semiconductor and in situ decorated Au NPs to form a PTCs@Au nanocomposite, which acted as an amplified signal tag and immune probe after the immobilization of Ab_2_ (Ab_2_-PTCs@Au). The prepared PTCs@Au with a Schottky heterojunction had an extended light absorption range and improved photon-to-electron conversion efficiency. When CEA existed, Ab_2_-PTCs@Au could bond to Ab_1_-modified photocathodes, achieving a stable and enhanced cathodic signal without the additional electron donor or acceptor, which also exhibited a low background and high sensitivity [107]. Moreover, the design of new detection modes will inject fresh vitality into the further development of photoelectrochemical sensing devices. Based on this, Wang et al. developed a photoelectric effect-driven multicolor visualized immunosensing platform using anti-CEA/Au NPs/Ag_2_S NPs@ZnO nanotubes (NTs)/FTO as photoelectrode and polyaniline/Prussian blue (PANI/PB) bilayer films as indicator electrodes [108]. Under light irradiation, the photoexcited Ag_2_S NPs@ZnO NTs generated electron–hole pairs in which photoelectrons and holes could migrate to reduce PB and oxidize PANI, respectively, enabling the multicolor transition of PANI/PB. When CEA was linked to Ag_2_S NPs@ZnO NT-based photoelectrodes, the increased steric hindrance inhibited the electron transport of photogenerated carriers so that the different color changes of PANI/PB were observed for quantitive detection of CEA. Similarly, Sun et al. designed a renewable dual-readout photoelectrochemical/visual immunosensor for synchronous CEA monitoring [109]. As shown in Figure 9, the designed photoelectrochemical sensing device was composed of two cells: a sensing cell for CEA detection and an electrochromic cell for reusage of PB. Among them, Ni:FeOOH/BiVO_4_ nanocomposite and PB served as the photoanode and cathode to constitute the sensing cell, respectively, in which the carried GOx, immobilized by CEA-driven sandwich-type immunoreaction in photoelectrochemical sensing cells, could catalyze glucose oxidation to generate H_2_O_2_ as a hole scavenger and then move to the photoanode for improved electron transfer efficiency. Simultaneously, the PB cathode could enable the photoelectron reduction of PB to Prussian white (PW) through a digital multimeter (DMM)-joined circuit, providing a synchronous naked eye visual/photoelectrochemical detection system. On the other hand, laccase-based biocathode could convert PW back to the original PB state via biocatalyzing oxygen reduction, achieving a renewable sensing system. Most strikingly, Hu’s group reported a single light-addressable photoelectrochemical immunosensor for multi-analyte detection (e.g., CEA and other tumor markers) [110], exhibiting a huge potential for future clinical diagnosis. Finally, an extended list of more currently photoelectrochemical CEA immunosensors are provided in this section (Table 3), together with their respective photoactive materials and analytical properties.

### 3.2. Photoelectrochemical Aptasensing

**Figure 10:**
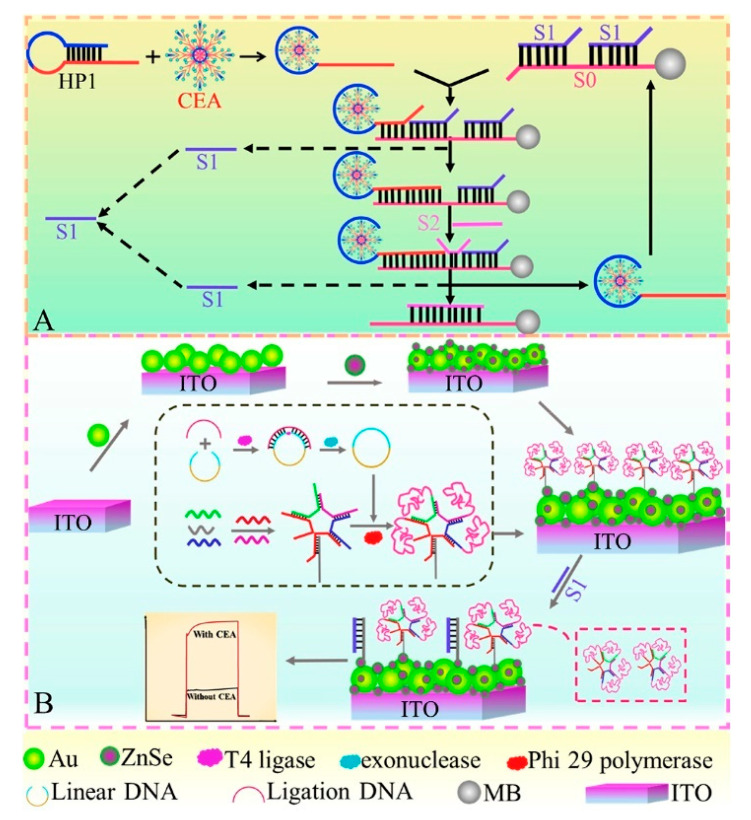
Schematic diagram of this proposed PEC biosensor for CEA determination. (**A**) Enzyme-free target cycling amplification strategy for generating S1; (**B**) Fabrication of the PEC “signal-off-on” biosensor based on ZnSe QDs/Au NPs and 3D DNA nanospheres. Reprinted with permission from ref. [124]. Copyright 2020 Elsevier.

Until now, since aptamers have the features of low immunogenicity, excellent programmability, and easy chemical modification, the focus has been on their sensing applications in clinical diagnosis, food security, and environmental monitoring [116,117,118]. Photoelectrochemical aptasensors, an emerging and interesting research subject, also make a significant contribution to the monitoring of tumor markers because of their selectivity, stability, and potential for miniaturization and portability [119,120]. Considering the biophysical binding between specific aptamers and CEA antigens, a series of photoelectrochemical aptasensors have been designed by various signaling strategies in recent years [121,122,123]. For example, Gao et al. constructed a signal-on photoelectrochemical aptasensing platform by self-assembly of 3D DNA nanospheres on Au NPs/ZnSe QDs-modified ITO electrodes [124]. As displayed in Figure 10, the formed DNA nanospheres were self-assembled by base complementary pairing and RCA and subsequently immobilized on the electrode’s surface for increased steric hindrance, making the photocurrent present “off” state. Meanwhile, the presence of target CEA could bind to its hairpin aptamer and then trigger multiple strand displacement processes to produce numerous single DNA strands (S1). After magnetic separation, the obtained S1 could competitively bind with captured DNA to remove DNA nanospheres on the electrode’s surface, so that the photocurrent signal switched to the “on” state, implementing the quantitative assay of CEA with the amplified photocurrent intensity. Subsequently, Yang et al. designed a polarity-reversal-mode photoelectrochemical CEA aptasensor using TiO_2_@AuNPs as negative and CdS QDs as positive signal indicators [125]. When CEA was added, a sandwich-type nanostructure was formed on captured CEA aptamer-modified TiO_2_@Au NPs in the presence of trigger CEA aptamers, which initiated the HCR process to immobilize CdS QDs on the electrode’s surface with the aid of CdS-labeled DNA probes. The formation of TiO_2_@Au NPs//CdS QDs plasmonic conformation contributed to the direct cathodic-to-anodic signal switch, making the detection limit of aptasensor decrease to 18.9 fg mL^−1^.

**Figure 11:**
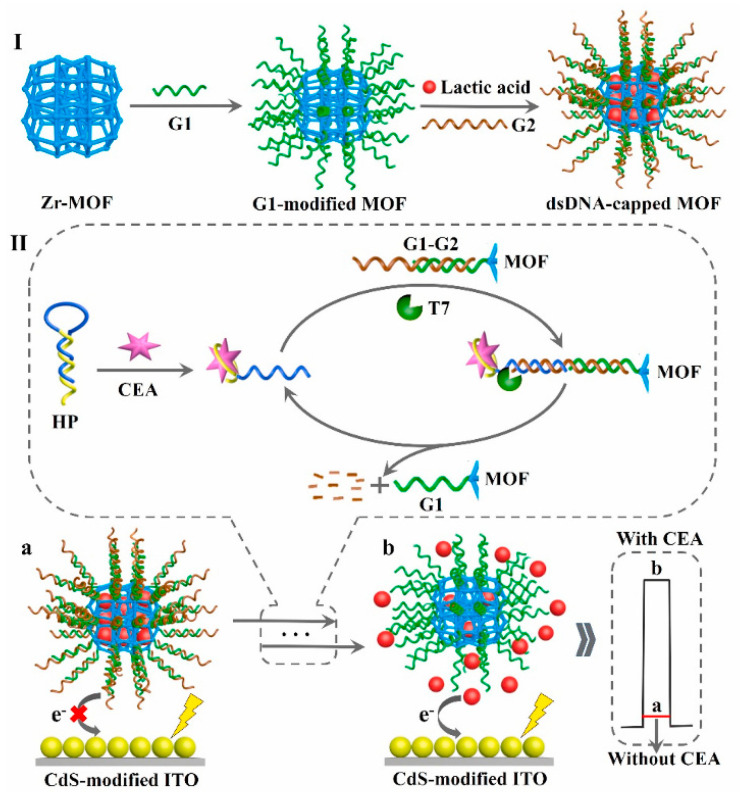
Schematic illustration of the proposed PEC biosensor for CEA determination. Reprinted with permission from ref. [127]. Copyright 2021 Elsevier.

To further improve the detection selectivity of sensing devices, a robust two-electrode aptasensor was designed by selecting ZnIn_2_S_4_ nanocrystal-decorated Fe^3+^-doped TiO_2_ nanotubes/Ti as photoanodes and CEA aptamer–bilirubin oxidase conjugate-modified Au NPs/carbon nanotubes/ITOs as photocathodes [126]. Among them, the photoanodes generated a stable photocurrent output, and the cathodic substrate presented excellent biorecognition and oxygen-reduction capability. Upon the addition of CEA, the aptamer–bilirubin oxidase conjugate departed due to the formation of a CEA–aptamer conjugate, and CEA determination hinged on a decline in the photocurrent response, endowing a desirable selectivity in the biological matrix because of the effective separation of biorecognition elements from the photoanodes. Moreover, to avoid time-consuming modification procedures, a sensitive immobilization-free aptasensor was developed using dsDNA-capped MOFs as electron donor encapsulations and CdS NPs as photoactive species [127]. As shown in Figure 11, the conformational structure of the self-blocked hairpin probe could change after the specific binding of its aptamer sequence to target CEA and then trigger the digestion reaction of T7 exonuclease-mediated recycling amplification due to the formation of duplex DNA, which effectively opened the core of dsDNA-capped MOFs to obtain abundant electron donors so that the photocurrent increased gradually with an increment in the target’s concentration, achieving a wide linear range (1.0 fg mL^−1^~10 ng mL^−1^) and low detection limit of 0.36 fg mL^−1^.

**Figure 12:**
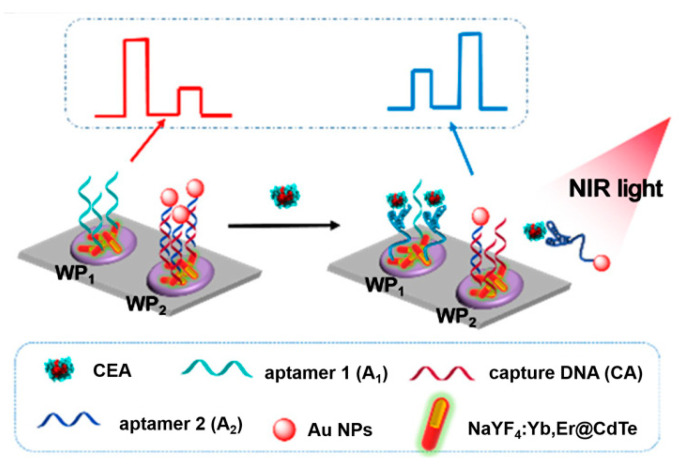
Construction of the up-conversion-mediated ratiometric PEC aptasensor for CEA detection. Reprinted with permission from ref. [129]. Copyright 2019 American Chemical Society.

Except for the above, several other ingenious aptasensors have been designed by the introduction of advanced nanoparticles and other technical characteristics. For instance, since a near-infrared excitation light source presents several characteristics, including near-zero photobleaching, low phototoxicity, and strong penetration depth, Tang et al. developed a near-infrared light-driven photoelectrochemical aptasensing of CEA in terms of HCR-dependent in situ generation of Ag_2_S NPs on dsDNA scaffold anchored on NaYF_4_:Yb,Er up-conversion NPs [128]. In the presence of CEA, an HCR process was initiated to produce a long dsDNA scaffold that allowed for the imbedding of large amounts of Ag^+^ ions via the chelation of C–Ag^+^–C. After reacting with sulfide, the generated Ag_2_S NPs could be excited by well-matched visible light emitted from up-conversion NPs under near-infrared light irradiation and, thus, an enhanced photocurrent was observed with an increasing CEA level. Later, to avoid to the signal’s background fluctuation, Tang’s group also designed a near-infrared light-stimulated and spatial-resolved ratiometric aptasensor for a CEA assay by combining with NaYF_4_:Yb,Er@CdTe nanocrystal-functioned dual channel electrodes and target-driven recognition events [129]. As displayed in Figure 12, CEA aptamer 1 and capture DNA:Au NP-labeled CEA aptamer 2 conjugate were anchored onto two adjacent photoelectrodes (WP_1_ and WP_2_), respectively. Upon the addition of CEA, aptamer 1 could bind to the analyte for the increased steric hindrance, whereas the specific reignition between aptamer 2 and CEA weakened the existing exciton–plasmon interactions with the release of the formed Au NP-labeled CEA aptamer 2@CEA conjugate, leading to an attenuated signal for WP_1_ and an enhanced signal for WP_2_. Obviously, the integration of various materials and detection modes can enable more possibilities for advanced aptasensing devices in clinical applications, and other interesting examples of recently published photoelectrochemical aptasensors for CEA analysis are listed in Table 4.

## 4. Conclusions and Future Perspectives

In this mini-review, we outlined the current progress of electrochemical and photoelectrochemical CEA biosensors, with a particular focus on the utilization of antibody- and aptamer-based recognition units. In addition to the intrinsic virtues of a small background, rapid response, and simple instrumentation, the specific target antibody/aptamer biorecognition reaction endows the developed electrochemical and photoelectrochemical biosensors with excellent anti-interference capability, and the successful fabrication of several advanced materials (e.g., MOFs and up-conversion nanoparticles) and amplified signaling tags (e.g., antibody–enzyme conjugates, aptamer–nanoparticles conjugates, and DNA assembly-based signaling probes) enables immunosensors and aptasensors with enhanced detection sensitivity. Furthermore, some robust detection modes, such as the dual-potential ratiometric assay and split-type sensing, can largely improve the accuracy and reliability of designed biosensors.

Despite substantial achievements in CEA assays, the existing electrochemical and photoelectrochemical biosensors are still confronted with some opportunities and challenges. For examples, the stepwise construction of a biosensing platform is generally time-consuming due to the tedious electrode assembly/washing procedures; thus, simplified and efficient construction methods are urgently needed for reliable and accurate CEA analysis. Moreover, modern biosensors are limited in point-of-care testing and real-time monitoring of CEA samples. To further expand their potential applications, the portable and family sized sensing devices should be explored, especially for photoelectrochemical biosensors that require excitation light, accompanied by the integration of paper-based electrodes or microfluidic devices. More interestingly, some emerging detection modes remain to be exploited for next generation of biosensors, such as self-powered and dual-signal-output-based biosensors, by coupling with various analytical methods (e.g., colorimetry and chemiluminescence) and other available techniques (e.g., biofuel cell and molecular imprinting), which will bring new vitality to the electrochemical and photoelectrochemical sensing devices in clinical diagnosis and medical research.

## Figures and Tables

**Table 1:** Performance comparison of recently reported electrochemical immunosensors for CEA.

Nanomaterial	Detection Technique	Linear Range (pg mL^−1^)	Detection Limit (pg mL^−1^)	Reference
MoS_2_-PBNCs	DPV	5–10,000	0.54	[25]
AuNPs/BSNa-CNC-PPy	SWV	0.001–200,000	0.00006	[26]
AuNPs/CNOs/SWCNTs/CS	SWV	0.1–400,000	0.1	[27]
AuNPs/PB-PEDOT	DPV	50–40,000	10	[28]
CPS@PANI@Au	DPV	6–12,000	1.56	[29]
CuFe-MoC@NG@PDA	i–t	0.01–80,000	0.003	[30]
AuNPs@ZrHCF@Fe_3_O_4_	SWV	0.5–50,000	0.15	[31]
Au/PDA/Au-PB/CNT	DPV	5–50,000	3.3	[32]
CNTs/rGO/Ag@BSA/PEDOT	LSV	2–50,000	0.1	[33]
Ag/MoS_2_/rGO	i–t	0.01–100,000	0.0016	[34]
Au/γ-PGA-DA@CS	EIS	0.02–20,000	0.01	[35]
HMSNs-Cu^2+^@HA	i–t	0.01–40,000	0.0035	[36]

PBNCs, Prussian blue nanocubes; BSNa, sodium benzenesulfonate; CNC, cellulose nanocrystalline; PPy, polypyrrole; CNOs, carbon nano-onions; SWCNTs, single-walled carbon nanotubes; CS, chitosan; PEDOT, poly(3,4-ethylenedioxythiophene); CPS, carboxy-functionalized polystyrene spheres; PANI, polyaniline; NG, N-doped graphene; PDA, polydopamine; ZrHCF, zirconium hexacyanoferrate; rGO, reduced graphene oxide; BSA, bovine serum albumin; γ-PGA, poly(γ-glutamic acid); HMSNs, hollow mesoporous silica nanoparticles; HA, hyaluronic acid.

**Table 2:** Performance comparison of recently reported electrochemical aptasensors for CEA.

Amplification Strategy	Detection Technique	Linear Range (pg mL^−1^)	Detection Limit (pg mL^−1^)	Reference
Strand displacement amplification	SWV	100–50,000	20	[78]
G-quadruplex/hemin DNAzyme and hybridization chain reaction	DPV	0.1–50,000	0.0182	[77]
Exonuclease III-assisted amplification	DPV	100–200,000	0.4	[79]
G-quadruplex/hemin DNAzyme	DPV	0.01–200,000	0.0032	[80]
Glucose oxidase and G-quadruplex/hemin DNAzyme-initiated cascade amplification	EIS	0.05–20,000	0.023	[81]
Hybridization chain reaction	EIS	0.1–40,000	0.03	[82]
Mg^2+^-dependent DNAzyme	DPV	0.001–1.5	-	[83]
Tetrahedral DNA and catalytic hairpin assembly	DPV	1–30,000	0.04567	[84]

**Table 3:** Analytical performances of various photoelectrochemical immunosensors for the determination of CEA.

Nanomaterial	Linear Range (pg mL^−1^)	Detection Limit (pg mL^−1^)	Reference
AuNPs/ZnO/Cu_2_O NWs	1.0–100,000	0.36	[111]
C_3_N_4_-BiOCl	0.1–10,000	0.1	[88]
AuNP-P5FIn/erGO	0.5–50,000	0.14	[112]
TiO_2_/C@ZnCdS MSDCs/Au	0.05–500,000	0.00228	[113]
I-BiOCl/CdS	10–40,000	2.0	[114]
Au/WS_2_ NTs	1.0–40,000	0.5	[90]
TiO_2_/CdS:Mn	0.1–100,000	0.02	[115]

NWs, nanowires; AuNP-P5FIn/erGO, AuNPs-decorated poly(5-formylindole)/electrochemically reduced graphene oxide nanocomposite; C@ZnCdS MSDCs, porous hollow carbon nanobubbles@ZnCdS multi-shelled dodecahedral cages; Au/WS_2_ NTs, AuNP-modified WS_2_ nanosheets; TiO_2_/CdS:Mn, Mn^2+^-doped CdS-modified TiO_2_ nanoparticles.

**Table 4:** Analytical performances of various photoelectrochemical aptasensors for CEA assays.

Nanomaterial	Linear Range (pg mL^−1^)	Detection Limit (pg mL^−1^)	Reference
PEDOT/Bi_2_S_3_/ZnO	1.0–100,000	0.67	[130]
g-C_3_N_4_/CuInS_2_	20–40,000	5.2	[131]
ZIS/Fe:TiO_2_	0.05–1000	0.018	[126]
NaYF_4_:Yb,Tm@ZnO	100–300,000	32	[132]
Tremella-like Bi_2_WO_6_	0.01–10,000	0.0026	[133]
BiFeO_3_	5.0–50,000	1.5	[134]
ZnO/g-C_3_N_4_-AuNPs	10–2500	1.9	[135]

g-C_3_N_4_/CuInS_2_, copper indium disulfide-sensitized graphitic-like carbon nitride; ZIS/Fe:TiO_2_, ZnIn_2_S_4_ nanocrystal-functionalized Fe^3+^-doped TiO_2_; BiFeO_3_, bismuth ferrite; ZnO/g-C_3_N_4_-AuNPs, g-C_3_N_4_-AuNPs-functionalized ZnO flower-rods.
